# Mechanism of the Inhibitory Effects of *Eucommia ulmoides* Oliv. Cortex Extracts (EUCE) in the CCl_**4**_-Induced Acute Liver Lipid Accumulation in Rats

**DOI:** 10.1155/2013/751854

**Published:** 2013-08-20

**Authors:** Chang-Feng Jin, Bo Li, Shun-Mei Lin, Raj-Kumar Yadav, Hyung-Ryong Kim, Han-Jung Chae

**Affiliations:** ^1^Department of Pharmacology and Institute of Cardiovascular Research, School of Medicine, Chonbuk National University, Chonbuk, Jeonju 561-180, Republic of Korea; ^2^Department of Dental Pharmacology and Wonkwang Biomaterial Implant Research Institute, School of Dentistry, Wonkwang University, Chonbuk, Iksan 570-749, Republic of Korea

## Abstract

*Eucommia ulmoides* Oliv. (EU) has been used for treatment of liver diseases. The protective effects of *Eucommia Ulmoides* Oliv. cortex extracts (EUCE) on the carbon tetrachloride- (CCl_4_-) induced hepatic lipid accumulation were examined in this study. Rats were orally treated with EUCE in different doses prior to an intraperitoneal injection of 1 mg/kg CCl_4_. Acute injection of CCl_4_ decreased plasma triglyceride but increased hepatic triglyceride and cholesterol as compared to control rats. On the other hand, the pretreatment with EUCE diminished these effects at a dose-dependent manner. CCl_4_ treatment decreased glutathione (GSH) and increased malondialdehyde (MDA) accompanied by activated P450 2E1. The pretreatment with EUCE significantly improved these deleterious effects of CCl_4_. CCl_4_ treatment increased P450 2E1 activation and ApoB accumulation. Pretreatment with EUCE reversed these effects. ER stress response was significantly increased by CCl_4_, which was inhibited by EUCE. One of the possible ER stress regulatory mechanisms, lysosomal activity, was examined. CCl_4_ reduced lysosomal enzymes that were reversed with the EUCE. The results indicate that oral pretreatment with EUCE may protect liver against CCl_4_-induced hepatic lipid accumulation. ER stress and its related ROS regulation are suggested as a possible mechanism in the antidyslipidemic effect of EUCE.

## 1. Introduction

*Eucommia ulmoides *Oliv. (EU) is one of the most popular tonic herbs in Asia. In a traditional herbal prescription, EU is used either as a single herb or in combination with one or two of the other herbs [1]. EU is also a popular folk drink and is used as a functional food reinforcing the muscles and lungs, lowering blood pressure, preventing miscarriages, improving liver and kidney tone, and increasing longevity [2]. EU, prepared from leaves or bark, is commonly used as liver and kidney tonic, thus, improving detoxification and circulation by the liver [3] and kidney [4, 5], respectively. EU leaves have been used for treatment of hepatic lipid accumulation [6, 7] and hepatic damage [3]. Recently, it was reported that *Eucommia ulmoides *Oliv. cortex extracts (EUCE) contain the same components as EU leaves, which have been the focus of medical research [2]. Studies have shown that EU leaf extracts have potent protective effects in various lipid peroxidation models and reduce oxidative damage of biomolecules [3, 8–11].

Hepatic accumulation of triglyceride (steatosis) is a major complication associated with obesity, insulin resistance, and alcoholic and nonalcoholic fatty liver disease [12]. This is because of increasing lipogenesis and decreasing *β*-oxidation followed by lipid peroxidation and mitochondrial dysfunction [12]. If left untreated, benign steatosis can develop into steatohepatitis, fibrosis, or cirrhosis.

Carbon tetrachloride (CCl_4_) is a colorless liquid that was commonly used as an anesthetic in the 19th and early 20th century. However, CCl_4_ was banded after establishment of its hepatotoxicity in the first 25 years of the 20th century. CCl_4_-induced liver damage in rats is the best characterized animal model of xenobiotic-induced free radical-mediated liver diseases [13]. Depending on the dose and duration, the effects of CCl_4_ on hepatocytes are manifested histologically as hepatic steatosis, fibrosis, hepatocellular death, or carcinogenicity [14]. Triglyceride secretion depends on the function of endoplasmic reticulum (ER) which assembles and secrets apolipoproteins in the liver. If ER function is damaged, secretion of apolipoproteins such as apolipoprotein B (ApoB) is inhibited, leading to hepatic lipid accumulation [15]. After oral administration, CCl_4_ concentrates in the liver, resulting in rapid accumulation of triglycerides in the liver [15]. Recently, CCl_4_ was shown to induce reactive oxygen species (ROS) through activation of cytochrome P450, leading to ER stress-mediated dysfunction. CCl_4_ is transformed to trichloromethyl free radical (CCl_3_OO^•^) by cytochrome P450 enzymes. Specifically, P450 2E1 interacts with NADPH-dependent cytochrome P450 reductase (NPR). Electron uncoupling between NPR and P450 2E1 is a major source of ROS on the ER membrane. ROS attack polyunsaturated fatty acid portions of membrane lipids to propagate a chain reaction, leading to lipid peroxidation and disruption protein synthesis, which results in the accumulation of proteins in the ER lumen and induction of ER stress [16]. It has been reported that severe and prolonged ER stress causes the accumulation of free radicals and disruption of protein secretion, leading to alteration of pathological conditions [17]. Regulation of ER stress has been suggested as one of the therapeutic/preventive approaches for the treatment of pathological conditions/diseases with ER stress [18]. Lysosomes are membrane-enclosed organelles that contain acid hydrolase enzymes. Lysosomal enzymes are known to play a role in regulating the ER stress response [19]. Proteins accumulated during ER stress are degraded by lysosomal enzymes through the endoplasmic reticulum-associated degradation (ERAD) pathway [20]. The lysosome-induced ERAD pathway has been suggested as one of the regulatory mechanisms of ER stress because the lysosomal activation can relieve intra-ER unfolded protein folding requirement.

ER stress regulation can be one of the potential mechanisms for ROS-associated hepatic steatosis. The secretion of ApoB is also altered in the presence of ER stress [21, 22]. CCl_4_-induced steatosis is related to ER stress and its related dysfunctions such as the alteration of apolipoproteins and ROS accumulation. Accordingly, the aim of this study is to investigate the effect of EUCE in CCl_4_-induced hepatic steatosis and ER stress. This study suggests that the preventive/therapeutic effect of the cortex extracts is due to the regulation of ER stress through lysosomal activation.

## 2. Materials and Methods

### 2.1. Materials****

*Eucommia ulmoides *Oliv. cortex was purchased from Sam-Hong Company (Seoul, Korea). Carbon tetrachloride (CCl_4_) and oil red O were purchased from Sigma-Aldrich Company (MO, USA).

### 2.2. Preparation of Plant Extracts

Dried cortex of *Eucommia ulmoides *Oliv. was authenticated in the Department of Pharmaceutical Chemistry of Yonsei University, Korea. The cortex was ground into a powder, mixed with extraction solvent (25% ethanol) at the ratio of 1 : 12, and then incubated in a 70°C water bath for 2 hours. Following incubation, the extraction solution was filtered, evaporated, and then dried to a powder by freeze drying at −55°C under low pressure. 

### 2.3. Treatment of Animals

Forty-eight Sprague Dawley (SD) male rats weighing 240–250 g (8-week-old) were purchased from Samtako Inc. (Osan, Korea) and housed in an air-conditioned room at 22 ± 2°C with a 12 h light/dark cycle. Animals were fed with rodent chow and tap water *ad libitum*. To study the protective effect against the CCl_4_-induced acute liver lipid accumulation, all rats were randomly divided into six groups of eight rats each: (A) control group, (B) CCl_4_ group, (C) CCl_4_ + EUCE 0.25 g/kg, (D) CCl_4_ + EUCE 0.5 g/kg, (E) CCl_4_ + EUCE 1 g/kg, and (F) EUCE 1 g/kg. Rats of groups B, C, D, and E were intraperitoneally injected with 1 mg/kg CCl_4_ mixed in olive oil, and rats of groups A and F were intraperitoneally injected with the same volume of pure olive oil. Rats in groups C, D, E, and F were treated with EUCE 8 times (twice/day for four days) before the injection of CCl_4_ (except for rats in group F). Four hours after CCl_4_ injection, each rat was anesthetized, blood was drawn, and liver tissues were removed. Blood samples were collected for ALT, AST, TG, and TC assays. Livers were excised from the animals and assayed for GSH levels, MDA formation, and pathological histology, according to the procedures described below. All experimental procedures were conducted in accordance with the National Institutes of Health. This experiment was approved by the Institutional Animal Care and Use Committee of Chonbuk National University, Jeonju, Korea.

### 2.4. Histological Staining

Liver samples were fixed in 3.7% formalin and dehydrated with 20% and 30% sucrose. Then, liver samples were embedded in OCT compound and cut into 10 *μ*m sections for oil red O staining. Liver sections were fixed in 3.7% formalin for 5 minutes washed with 60% isopropanol. Fixed samples were then stained with 0.3% oil red O in 60% isopropanol for 30 min and washed with 60% isopropanol. Sections were counterstained with hematoxylin, washed with running water for 5 min, and mounted with an aqueous solution. Stained sections were quantified by histomorphometry.

### 2.5. DPPH Radical Scavenging Assay

Free radical scavenging activity of the EUCE was measured using the 1, 1-diphenyl-1-picrylhydrazyl (DPPH) radical scavenging assay [23]. EUCE solution (0.3 mL) at a range of concentrations was mixed with 0.2 mM DPPH in methanol (2.7 mL). The mixture was shaken vigorously and allowed to stand for 1 h before the absorbance was measured at 517 nm. Free radical scavenging activity was calculated as the following percentage: [(*A*_*s*_ − *A*_*i*_)/*A*_*s*_]×100 (*A*_*s*_ is absorbance of DPPH alone and *A*_*i*_ is absorbance of DPPH in the presence of various extracts). Butylated hydroxyl toluene (BHT) at a concentration identical to the experimental samples was used as a reference.

### 2.6. Biochemical Determination

Serum levels of triglyceride (TG), total cholesterol (TC), alanine transaminase (ALT), aspartate aminotransferase (AST), liver glutathione (GSH), and malondialdehyde (MDA) were determined using a commercial analysis kit obtained from the ASAN Institute of Biotechnology (Seoul, Korea) and Jiancheng Institute of Biotechnology (Nanjing, China). Hepatic concentrations of TC and TG were also measured after chloroform-methanol extraction. Liver samples (115 mg) mixed with 500 *μ*L D-PBS were homogenized and centrifuged at 3500 g for 5 minutes. The supernatants were removed and centrifuged briefly after the addition of 400 *μ*L chloroform-methanol (1 : 2). Then, 250 *μ*L chloroform and 250 *μ*L water were added, and the samples were centrifuged at 3000 rpm for 5 minutes. The lower phase was transferred to a new tube, and residual chloroform was evaporated by heating at 55°C. After chloroform evaporation, 25 *μ*L of RIPA buffer was added and the samples were resuspended by heating at 90°C for 3 minutes. TG and TC levels were then measured with commercial kits.

### 2.7. Western Blot Analysis

Proteins were separated under nonreducing conditions, transferred to nitrocellulose membranes, and incubated for 2 h at room temperature in blocking buffer (20 mM Tris, pH 7.5, 137 mM NaCl, 0.1% Tween 20, and 5% nonfat dry milk). Blots were washed three times and incubated overnight at 4°C in the same buffer containing 0.5% dry milk and primary antibody (1 : 1000 dilution). The blots were then washed and incubated with mouse horseradish peroxidase-conjugated secondary antibody (1 : 4000) in 1.0% skim milk for 1 hour at room temperature. Immune reactivity was detected by chemiluminescence. Then, the intensities of band were measured and quantified as described by Luke Miller (http://lukemiller.org/index.php/2010/11/analyzing-gels-and-western-blots-with-image-j/).

### 2.8. Measurement of P450 2E1 Activity

Specific activity for P450 2E1 was evaluated in liver homogenates utilizing model substrates. P450 2E1 catalyzes the hydroxylation of *p*-nitrophenol to *p*-nitrocatechol [24]. First, we isolated liver microsomes. Then, microsomal proteins were incubated in assay buffer (1 mM ascorbic acid, 2 mM magnesium chloride, 1 mM NADPH, and 50 mM phosphate buffer, pH 6.8) at 37°C in a shaking water bath. Following incubation, the P450 2E1 reaction was stopped on ice with addition of 20% trichloroacetic acid. Samples were concentrated to 50 *μ*L, and then the supernatants were transferred to a 96-well plate. Prior to reading, 2 M NaOH was added to each sample or standard. Absorbance was measured at *λ* = 517 nm on a 96-well plate reader [25]. 

### 2.9. Measurement of Lysosomal Enzymes Activity

Lysosomes were isolated from liver tissues for the measurement of lysosomal enzymes activity. Isolation of lysosomes was performed using a method based on differential and density-gradient centrifugation techniques [26]. After isolation of lysosomes, the 100 *μ*L assay mixtures consisted of the following: *β*-galactosidase assay, 0.5 mM 4-methylumbelliferyl- (MU-) *β*-galactoside in 100 mM citrate-phosphate buffer (pH 4.35) with 0.4 M NaCl; *β*-glucuronidase assay, 1 mM 4-*β*-glucuronide in 100 mM acetate buffer (pH 4.0); *α*-mannosidase assay, and 4 mM *α*-mannopyranoside in 100 mM acetate-phosphate buffer (pH 4.0). Assay mixtures were incubated at 37°C for 30 minutes with an excitation wave length from 360 nm to 410 nm.

### 2.10. Statistical Analysis

All data are expressed as mean ± SD. All comparisons were done by one-way analysis of variance (ANOVA) with Dunn's test for post hoc analysis. *P*values < 0.05 were considered statistically significant.

## 3. Results

### 3.1. Effect of EUCE on CCl_**4**_-Induced Histological Changes in the Liver

**Figure 1:**
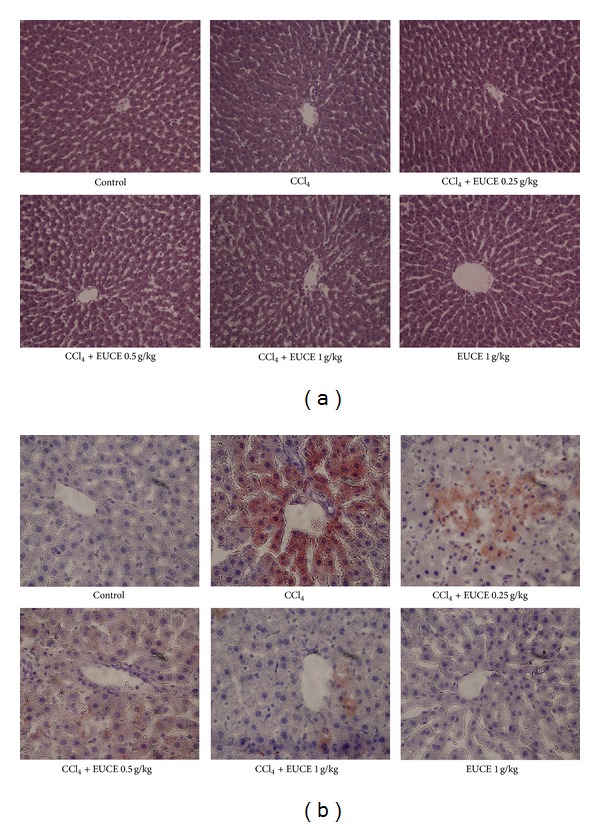
Effect of EUCE on CCl_4_-induced histological changes in liver. Rats were injected with 1 mg/kg CCl_4_, and livers were isolated after 4 hours. Representative photomicrographs (200x) of liver sections from rats (*n* = 8) stained with hematoxylin and eosin (a) and oil red O (b) are shown.

Liver tissues were collected to assess the effect of EUCE on liver pathological changes. As shown in Figure 1(a), H&E staining of liver sections demonstrated normal liver architecture in CCl_4_-treated rats. Oil red O staining showed the development of acute lipid accumulation in rats 4 hours after injection of CCl_4_, whereas no histological abnormalities were observed in normal control or EUCE-treated rats (Figure 1(b)). Administration of EUCE prevented fatty deposition in hepatocytes. As demonstrated by histological results, H&E staining indicated no morphology changes in the CCl_4_-treated rats compared with the control group, while oil red O staining showed that CCl_4_-induced lipid accumulation was blocked by pretreatment with EUCE. Specifically, a significantly reduced in lipid accumulation was observed with administration of EUCE at a dose of 1 g/kg.

### 3.2. Effect of EUCE on Lipid Metabolism

**Figure 2:**
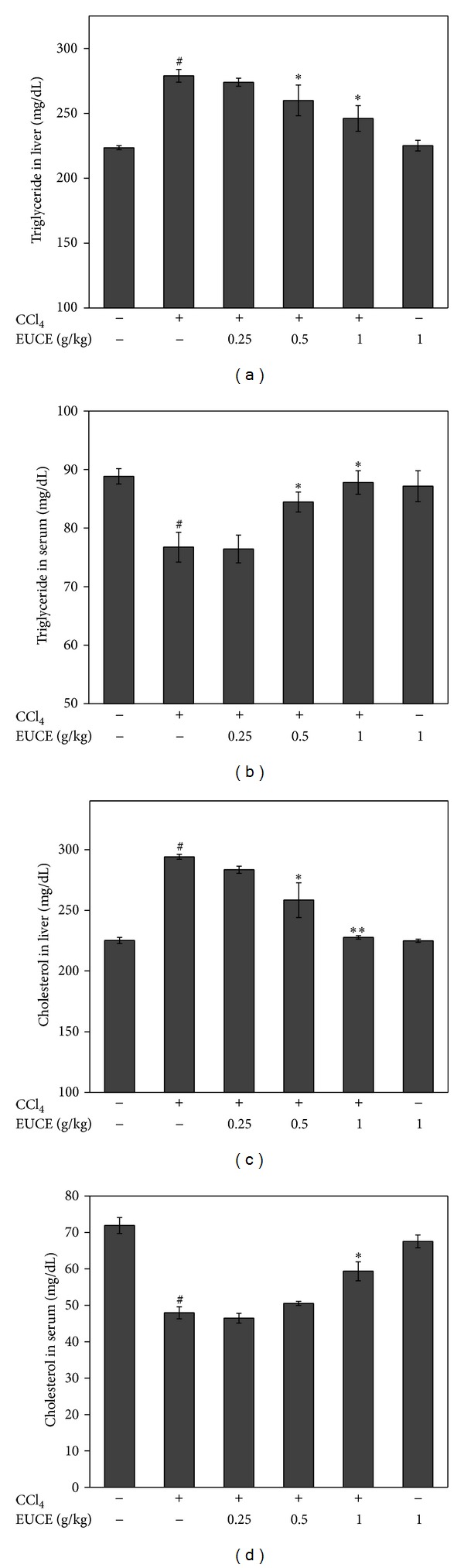
Effect of EUCE on CCl_4_-induced inhibition of hepatic triglyceride and cholesterol secretion. Rats were injected with 1 mg/kg CCl_4_, and livers were isolated after 4 hours. Triglyceride and cholesterol levels were measured in the liver (a), (c) and plasma (b), (d), respectively. Values are mean ± SD, *n* = 8. Asterisks indicate differences from the group treated with CCl_4_ only (*P < 0.05; **P < 0.001). ^#^ *P* < 0.05 indicates a significant difference compared with the control group.

To analyze the possible role of EUCE in lipid metabolism, which plays a major role in fatty liver formation, triglyceride (TG) and total cholesterol (TC) in liver and serum were investigated. As shown in Figure 2, TG and TC levels were significantly increased by acute CCl_4_ injection, and this was blunted by EUCE pretreatment. The data suggested that EUCE may regulate acute lipid accumulation.

### 3.3. Effect of EUCE on Free Radical Scavenging Activity and Hepatic GSH Levels

**Figure 3:**
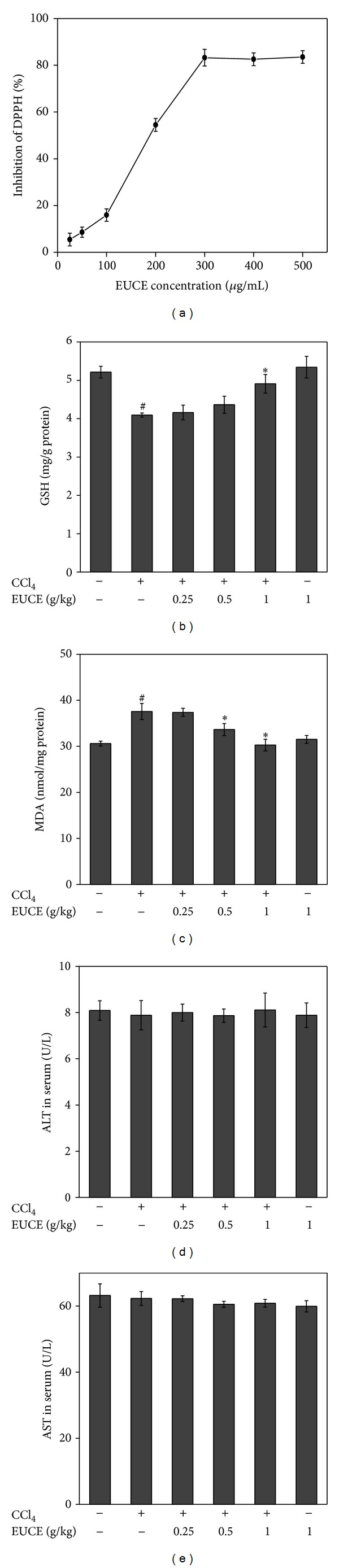
Effect of EUCE on free radical scavenging activity and CCl_4_-induced increases in liver glutathione (GSH) and malondialdehyde (MDA). A difference was found in ALT and AST levels after injection of CCl_4_. Values are mean ± SD, *n* = 8. Asterisks indicate differences from the group treated with CCl_4_ only (*P < 0.05). ^#^ *P* < 0.05 indicates a significant difference compared with the control group.

The effect of EUCE on DPPH free radical scavenging activity was tested, and the results are presented in Figure 3(a). As shown in Figure 3(a), IC_50_ values of EUCE were about 310 *μ*g/mL. EUCE exhibited a curve of antioxidant activity. Glutathione (GSH) constitutes the first line of defense against free radicals [27]. GSH levels were significantly depleted by CCl_4_ administration; however, depletion of GSH induced by CCl_4_ was significantly reversed in a dose-dependent manner by pretreatment with EUCE (Figure 3(b)).

### 3.4. Effect of EUCE on CCl_**4**_-Induced Lipid Peroxidation Levels

CCl_4_-induced ROS accumulation has been associated with the pathology status induced by CCl_4_ [28]. We investigated MDA content which is a result of lipid peroxidation by ROS. Compared with the control group, the CCl_4_-treated group showed significantly increased MDA content (Figure 3(c)). However, EUCE treatment at dose of 0.5 and 1.0 g/kg significantly decreased MDA content. These results indicate that EUCE has the potential to reduce lipid peroxidation induced by CCl_4_. Furthermore, ALT and AST, hepatic enzymes that are released into the blood stream by liver damage, were not increased (Figures 3(d) and 3(e)). Thus, liver function was not affected during the transient time periods.

### 3.5. Effect of EUCE on ApoB and ApoA1 Levels in the Liver

**Figure 4:**
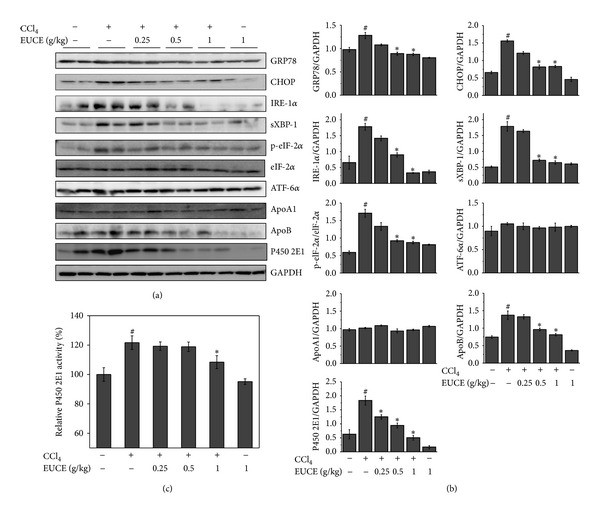
Effect of EUCE on CCl_4_-induced ER stress response and expression of ApoB and P450 2E1. Rats were injected with 1 mg/kg CCl_4_, and livers were isolated after 4 hours. (a) Western blotting was performed on liver protein extracts with anti-GRP78, CHOP, p-eIF-2*α*, eIF-2*α*, ATF-6*α*, IRE-1*α*, sXBP-1, ApoA1, ApoB, P450 2E1, or GAPDH. (b) Graphs showing quantification of (a). (c) P450 2E1 activity was measured in liver. Asterisks indicate differences from the group treated with CCl_4_ only (*P < 0.05). ^#^ *P* < 0.05 indicates a significant difference compared with the control group.

Lipids are carried on apolipoproteins (Apo) in plasma [29]. ApoA1 is responsible for carrying HDL, and ApoB is responsible for carrying LDL and triglyceride [30]. As shown in Figure 4, the expression of ApoB was increased in the liver after 4 hr of exposure to CCl_4_, but ApoA1 was no affected. Increased expression of ApoB in the liver was suppressed by pretreatment with EUCE in a dose-dependent manner, particularly at a dose of 1 g/kg. Transient accumulation of triglyceride and cholesterol in liver induced by CCl_4_ occurred via decreased plasma ApoB production and VLDL secretion [15]. These results show that pretreatment with EUCE may improve ApoB secretion compared with CCl_4_-treated group.

### 3.6. Effect of EUCE on CCl_**4**_-Induced ER Stress and P450 2E1 Activation

Abnormal ER function affects secretion of apolipoproteins. A previous study reported on the identification of proteins that play an important role in the survival of liver cells after induction of ER stress by CCl_4_ [31]. As shown in Figures 4(a) and 4(b) in CCl_4_-treated rats, the expression of ER stress proteins GRP78, CHOP, IRE-1*α*, and spliced XBP-1 was increased, and eIF-2*α* was phosphorylated in liver tissue. Pretreatment with EUCE reduced the expression of ER stress proteins in a dose-dependent manner. P450 2E1, the major isozyme involved in the bioactivation of CCl_4_ and responsible for ER stress-induced ROS, was also increased in CCl_4_-treated rats. Consistently, pretreatment of EUCE significantly reduced the P450 2E1 activity (Figure 4(c)).

### 3.7. Effect of EUCE on Lysosomal Enzyme Activity

**Figure 5:**
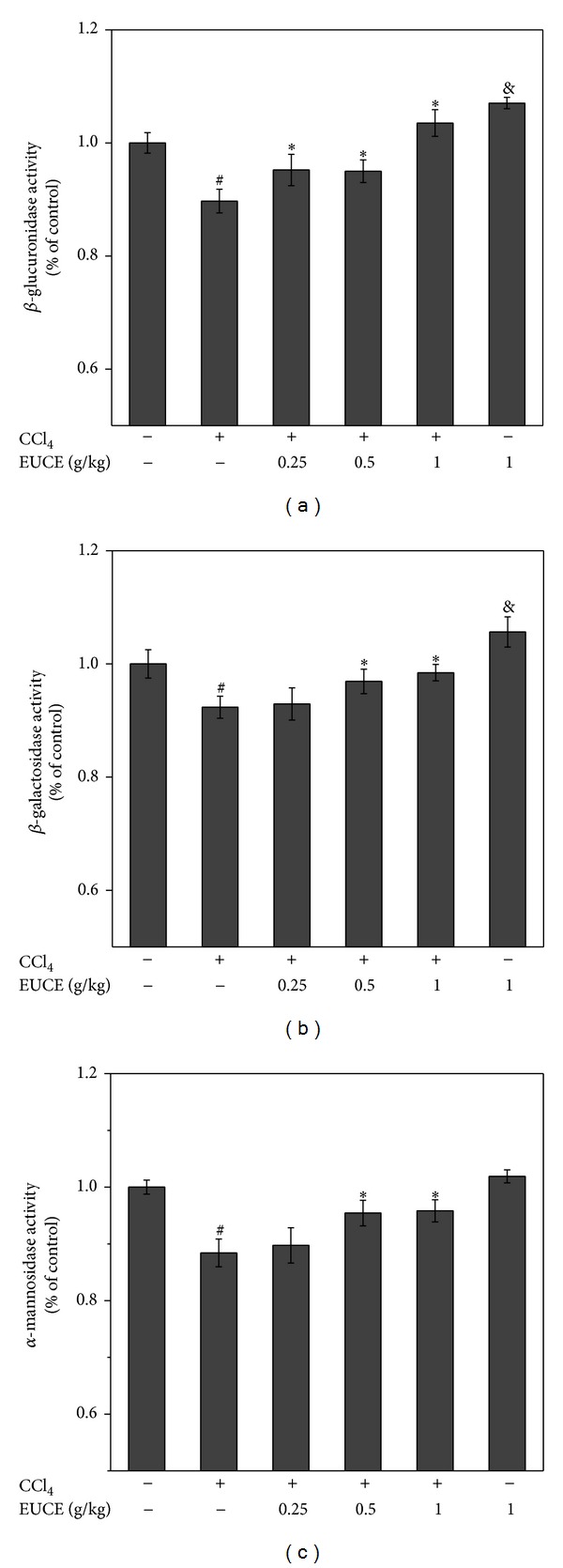
Effect of EUCE on CCl_4_-induced lysosomal enzymes activity. Rats were injected with 1 mg/kg CCl_4_, and livers were isolated after 4 hours. The lysosomal enzymes activity was measured by a kit. Values are mean ± SD, *n* = 8. Asterisks indicate differences from the group treated with CCl_4_ only (*P < 0.05). ^&^ *P* < 0.05, ^#^ *P* < 0.05 indicate a significant difference compared with the control group.

Enhanced activity of lysosomal enzymes has been suggested to have a regulatory role on ER stress. Pretreatment with EUCE significantly increased the activity of lysosomal enzymes compared with the control group, particularly at a dose of 1 g/kg (Figure 5), thus, indicating the potential role of EUCE in enhancing lysosomal enzyme activity.

## 4. Discussion

This study showed that rats pretreated with EUCE were protected against CCl_4_-induced hepatic lipid accumulation, as confirmed by histological observation and decreased levels of triglyceride and total cholesterol compared with the control group. EUCE regulated ER stress response by decreasing P450 2E1 activity and ROS accumulation. CCl_4_-induced ER stress response, enhancing P450 2E1 activity. In our previous study, we showed that ER stress and its related P450 2E1 activity play an important role in CCl_4_-induced steatosis in rats [32]. In the CCl_4_-induced hepatic steatosis, possible role of ROS accumulation has been suggested, ER stress and its consequent increase of P450 2E1, leading to ROS accumulation. A converse mechanism, P450 2E1 activation and ROS accumulation induced by CCl_4_, leading to ER stress, is not able to be ruled out. Although the cause or consequence of ROS production in relation to ER stress has not been clearly established, the pieces of evidence of CCl_4_-induced ROS production have been already accumulated. Recently, CCl_4_ was shown to induce hepatotoxicity by enhancing the formation of free radicals through their metabolism, leading to lipid peroxidation of cellular and organelle membranes as a primary pathogenic step [33].

In this study, P450 2E1 activity was significantly increased 4 hours after exposure to CCl_4_ compared with the control group, while the EUCE-pretreated group showed decreased P450 2E1 activity compared with the CCl_4_-treated group (Figure 4(c)). CCl_4_ is widely used for experimental induction of liver steatosis/cirrhosis in relatively acute settings [34]. Cytochrome P450 2E1, a member of the cytochrome P450 mixed-function oxidase system, can catalyze CCl_4_ to form trichloromethyl free radicals which interact with molecular oxygen to form trichloromethyl peroxy radicals [16, 35–37]. These free radicals play an important role in the pathogenesis of liver steatosis by binding to proteins or lipids, which then initiates lipid peroxidation [38]. It has also been reported that P450 2E1 is the primary enzyme responsible for low-dose carbon tetrachloride metabolism in human liver microsomes [39]. The hepatotoxic effects of CCl_4_ are dependent on the cosubstrate NADPH because conversion of CCl_4_ to CCl_3_OO^•^ occurs in conjunction with the NADPH-cytochrome P450 electron transport chain in the liver endoplasmic reticulum [13, 40]. Cytochrome P450 transfers an electron from NADPH to CCl_4_, causing CCl_4_ to be reduced to CCl_3_OO^•^ and Cl^•^. A previous study showed that the expression of P450 2E1 and its interaction with NPR both increase after CCl_4_ treatment; however, both were also shown to decrease after 12 hours [32]. 

This study also showed that EUCE pretreatment increased GSH level that had been lowered by CCl_4_. GSH helps prevent damage of important cellular components caused by reactive oxygen species such as free radicals and peroxides. Moreover, GSH plays a preventive/therapeutic role in CCl_4_-induced hepatic toxicity via the P450 2E1 pathway [37]. GSH is an antioxidant that contributes to the detoxification of CCl_4_, which induces hepatic lipid accumulation through its free radical derivatives [16]. As reported, increased production of ROS induced by CCl_4_ plays a role in liver steatosis/cirrhosis through two distinct pathways. One pathway involving P450 2E1 leads to the formation of toxic peroxyl and alkoxyl radicals that initiate lipid peroxidation. The second pathway involves a detoxification reaction that lowers GSH levels [41, 42]. Therefore, the DPPH assay was used in this study to evaluate the effect of EUCE on free radical scavenging activity. EUCE may play an important role in raising GSH levels [43]. In addition, oxidative stress, which is considered to play an important role in the development of hepatic steatosis/cirrhosis, is associated with lipid peroxidation and lower levels of GSH [44].

In addition, the CCl_4_-treated group had significantly increased levels of MDA, whereas the EUCE-pretreated group had significantly decreased levels of MDA in liver. MDA is a metabolite of the free radical-mediated lipid peroxidation cascade and therefore is used as a marker of lipid peroxidation. Thus, the biochemical mechanism underlying the development of CCl_4_ steatosis/cirrhosis may involve MDA. In CCl_4_-treated rats, significantly increased levels of MDA have been shown [45].

In this study, the expressions of ER stress proteins and hepatic ApoB were both increased in CCl_4_-treated rats, whereas they were decreased in the EUCE groups (Figures 4(a) and 4(b)). This result suggests that EUCE might modify ApoB synthesis and therefore could impact on liver and plasma triglyceride content. Apolipoproteins, lipid binding proteins that form lipoproteins to transport lipids through the lymphatic and circulatory systems, are regulated by normal function of the ER. With ER stress, protein folding and secretion can be significantly affected [46]. As reported, ApoB, a member of the apolipoprotein family, is reduced in CCl_4_-treated rats [47]. CCl_4_ decreases secretion of very low density lipoproteins and rapidly increases triglycerides in rat livers [48]. Consistently, in CCl_4_-treated groups, triglycerides rapidly accumulate in liver, contributing to the failure of secretory mechanisms [49]. It has been reported that decreased ApoB secretion is responsible for hepatic lipid accumulation [50, 51].

The results of lysosomal enzymes activity shown in Figure 5 indicate that EUCE increased the activity of lysosome enzymes by improving ER function. Through the protein degradation machinery activation, the requirement of protein folding can be relieved. GRP78, also known as binding immunoglobulin protein (Bip), is of a particular importance as a regulator of the ER stress response. GRP78 normally binds to three main transmembrane proteins: the protein kinase RNA- (PKR-) like ER protein kinase (PERK), activating transcription factor 6 (ATF-6), and inositol-requiring enzyme 1*α* (IRE-1*α*) [52]. In addition, GRP78 also serves as the master regulator of the ER stress response by binding and inactivating stress sensors at the luminal surface of the ER [53]. Initiation of ER stress response occurs when the quantity of misfolded or unfolded proteins in the ER exceeds the capacity of chaperone proteins that trigger the activation of UPR pathways. To reduce the accumulation of proteins in the ER, PERK phosphorylates eukaryotic initiation factor 2*α* (eIF-2*α*) to attenuate translation of proteins [54]. IRE-1*α* is related to genes involved in the transport of unfolded proteins out of the ER and in their degradation by ER-associated degradation (ERAD) pathway [55]. As reported, misfolded and unstable proteins in the ER are degraded by the ERAD pathway [56]. Lysosomes mediate degradation of the majority of intracellular proteins, and lysosomal activity is involved in the ERAD II pathway [57]. Through the stably maintained lysosomal activity, it may be suggested that EUCE regulates ER stress and its subsequent reduced bioactivation of CCl_4_ to CCl_3_OO^•^ by P450 2E1, which can activate ER stress in response.

In conclusion, the results of this study indicate that pretreatment with EUCE effectively decreases hepatic lipid accumulation induced by CCl_4_. EUCE increases lysosomal enzyme activity, relieving the protein folding requirement leading to the attenuation of ER stress. The regulatory effect of ER stress is suggested to improve ApoB secretion as well as to regulate the biotransformation of CCl_4_ and its resultant inhibition of ROS accumulation. Future research is necessary to unravel the mechanism of underlying the ability of EUCE to increase lysosomal enzyme activity, a suggested ER stress regulation mechanism.
